# 444. The disruptive effects of COVID-19 on the incidence of hospital acquired staphylococcus aureus bacteremia (HA-SAB) and its associated factors with mortality in a tertiary teaching hospital in an upper-middle income country

**DOI:** 10.1093/ofid/ofad500.514

**Published:** 2023-11-27

**Authors:** Sasheela Sri La Sri Ponnampalavanar, Sheron Sir Loon Goh, Arulvani Rajandra, Nur Alwani Suhaimi, Siti Norintan Zainon, Sazali Basri, Rong Xiang Ng, Yong Yaw Yeow, Anjanna Kukreja

**Affiliations:** University of Malaya, Kuala Lumpur, Kuala Lumpur, Malaysia; Universiti Malaya, Kuala Lumpur, Kuala Lumpur, Malaysia; Universiti Malaya, Kuala Lumpur, Kuala Lumpur, Malaysia; Universiti Malaya Medical Centre (UMMC), Kuala Lumpur, Kuala Lumpur, Malaysia; Universiti Malaya Medical Centre, Kuala Lumpur, Kuala Lumpur, Malaysia; University of Malaya Medical Centre, Kuala Lumpur, Kuala Lumpur, Malaysia; University of Malaya, Kuala Lumpur, Kuala Lumpur, Malaysia; Hospital Sungai Buloh, Kuala Lumpur, Kuala Lumpur, Malaysia; University of Malaya, Kuala Lumpur, Kuala Lumpur, Malaysia

## Abstract

**Background:**

Hospital acquired bacteremia is predominantly caused by *Staphylococcus aureus.* Given its virulence, patients may experience serious complications that can be difficult to diagnose at the time of the infection leading to increased morbidity and mortality. The objective of this study is to evaluate the incidence of hospital-acquired Staphylococcus aureus bacteremia (HA-SAB) and its risk factors associated with mortality among hospitalized patients in Universiti Malaya Medical Centre (UMMC), a tertiary teaching hospital in Malaysia

**Methods:**

This is a retrospective analysis of patients with HA-SAB admitted to UMMC over three time periods (P) in relation to the COVID-19 pandemic; P1 beginning of the pandemic (July 2020-May 2021), P2 during the peak of pandemic (June 2021-June 2022) and P3 recovery phase (July 2022-March 2023). We included patients ≥18 years old who fulfilled the criteria for HA-SAB. HA-SAB was defined as the first positive blood culture performed ≥48 hours after admission in patients with no evidence of infection at the time of admission. HA-SAB rates per 100 days admission and its risk factors associated with mortality were analyzed.

**Results:**

A total of 287 cases of HA-SAB were identified (P1=80, P2=151 and P3=56) and the overall mortality rate was 31%. The HA-SAB rate increased by 42% from 0.19 (P1) to 0.33 (P2) per 100 admission days and decreased to 0.15 per 100 admission days (P3). On multivariate analysis, risk factors associated with mortality among patients with HA-SAB were older age (OR: 1.04, 95% Cl: 1.02-1.06), use of arterial lines (OR: 4.30, 95% Cl:1.57-11.5) and secondary bloodstream infection (BSI) (OR: 0.39, 95% Cl: 0.22 - 0.70).

**Conclusion:**

The COVID-19 pandemic was associated with substantial increase in HA-SAB rate. This was likely due to changes in infection control measures in general and line care prevention bundle specifically to cope with the pandemic such as extended use of personal protective equipment (PPE), and non-adherence to the 5-moments of hand hygiene especially changing gloves between patients. Older age, use of arterial lines and secondary BSI were significantly associated with mortality among patients with HA-SAB.

**Disclosures:**

**All Authors**: No reported disclosures

